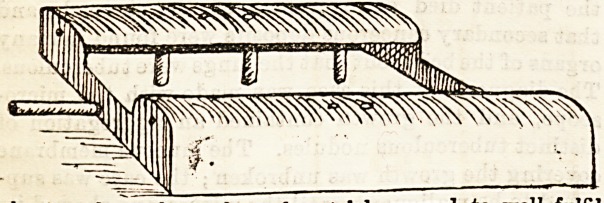# New Appliances and Things Medical

**Published:** 1896-01-11

**Authors:** 


### NEW APPLIANCES AND THINGS MEDICAL.

[We shall be glad to receive, at our Office, 428, Strand, London, W.C., from the manufacturers, specimens of all new preparations and appliances, which may be brought out from time to time.]

### DRESSING TRAY.

The accompanying illustration shows a dressing tray, which is the invention of Miss Marshall, Lady Superintendent of St. Monica's Home-Hospital for Sick Children, Brondesbury Park, and has been found of practical use in that institution for some months past. Many, if not most, of the little patients at St. Monica's are sufferers from hip disease, spinal affections, and other protracted illnesses, in which it is desirable that the dressing and irrigation of wounds shall be accomplished with as little movement and consequent pain to the patient as possible; and the particular form and shape of the tray here shown has, after trial, proved to well fulfil these conditions, and to meet with the approval of the doctors and all concerned. The tray is made of zinc, the curved side pieces being covered with thin macintosh pads, on which the child is placed, with a pillow to support the shoulders. A tube is attached to the nozzle seen in the drawing to carry away fluids into a pail or basin. In this way, in cases of psoas abscess, excision of hip, &c., the dressings can be removed, and the wounds irrigated and redressed with a minimum of movement. These dressing-trays can be obtained from the Lady Superintendent at the Home; the price is 7s. 6d.

### A NEW FOOD—NUTROA.

(Nutroa Co., Limited, 54, Chiswell Street, London, E.C.)

The Nutroa Company is now preparing some new food preparations which should demand the attention of all those who are interested in dietetics. The object in view has been the manufacture of an ideal food both for children and invalids. Nutroa, besides having digestive properties of its own, may be added to ordinary flour, and considerably increases its nutritional value by replacing those valuable nitrogenous portions of the wheat which are removed by the modern process of milling. Nutroa food has been shown by analysis to contain the exact proportions of proteids, carbohydrates, and fats, which physiologists have proved over and over again to be requisite for the maintenance of life. Chemically speaking it is a perfect food for infants. Chemistry cannot show, however, whether a theoretically perfect food will in practice answer all requirements. Not only is it quantitatively adjusted to the demands of infant life, but it is readily assimilated and pleasant to taste. By the addition of fresh milk it possesses those anti-scorbutic properties for want of which many artificial foods are directly responsible for the production of scurvy and rickets in children. We have only one suggestion to the manufacturers, and that is to make it quite clear in the printed directions how much of the ready prepared food corresponds to the usual quantity of human milk necessary for infants of different ages. An infant of a week old should have the food much more diluted with water than the casual reader would be led to suppose from reading the "directions for use." When this little alteration has been made we should feel justified in recommending it as a perfectly safe and valuable food for nursery use.

## Figures and Tables

**Figure f1:**